# SENSORIMOTOR IMPAIRMENTS AND DEMENTIA: A CROSS-SECTIONAL ANALYSIS OF NATIONAL HEALTH AND AGING TRENDS STUDY

**DOI:** 10.1093/geroni/igad104.0545

**Published:** 2023-12-21

**Authors:** Anis Davoudi, Yuri Agrawal, Alden Gross, Amal Wanigatunga, Nicholas Reed, Joshua Ehrlich, Jennifer Schrack

**Affiliations:** Johns Hopkins University, Baltimore, Maryland, United States; Johns Hopkins School of Medicine, Baltimore, Maryland, United States; Johns Hopkins University Bloomberg School of Public Health, Baltimore, Maryland, United States; Johns Hopkins School of Public Health, Baltimore, Maryland, United States; Johns Hopkins Bloomberg School of Public Health, Baltimore, Maryland, United States; University of Michigan, Ann Arbor, Michigan, United States; Johns Hopkins University, Baltimore, Maryland, United States

## Abstract

Sensory impairments in vision and hearing are linked to dementia in older adults. However, this association has not been investigated in conjunction with motor function performance. We examined cross-sectional sensory and motor function and dementia status data from 1,765 participants in the National Health and Aging Trends Study (NHATS R11, 2021) (32.7% aged ≥80 years; 55% women) who completed objective physical function and sensory testing. Motor function was defined using grip strength (kg), gait speed (m/s), standing balance, and chair stand speed (stands/sec). Sensory function was defined using hearing (pure tone average for better ear (BPTA) (dB)) and vision (contrast sensitivity (logCS)). Dementia was categorized as possible or probable using NHATS dementia classification. Survey-weighted logistic regression models estimated the likelihood of dementia by impairment in sensory and motor function, adjusted for demographic and medical conditions. 185 (10.5%) participants had possible or probable dementia. In models incorporating both motor and sensory impairments, impaired standing balance (<3 standing balance score in SPPB) and slow walking speed (<0.8m/s) were associated with higher odds of dementia (OR=1.75; 95%CI 1.10-2.79; and 2.39 95%CI 1.37-4.15, respectively), while hearing impairment (BPTA>25dB) and contrast sensitivity (<1.55logCS) were not associated with higher prevalence of dementia. In this nationally representative sample of older US adults, results suggest motor function is more strongly associated with dementia relative to sensory function among participants able to complete physical function testing. Future research is needed to understand how the associations of sensory and motor function with dementia change from mid-to-late life.

